# Reducing CIED-Related Morbidity: “LESS Is More”

**DOI:** 10.3390/jcm11164782

**Published:** 2022-08-16

**Authors:** François D. Regoli

**Affiliations:** 1 Service of Cardiology, Regional Hospital of Bellinzona and Valleys (ORBV), Via Ospedale 12, CH-6500 Bellinzona, Switzerland; francoisdiederik.regoli@eoc.ch; 2 Division of Cardiology, Cardiocentro Ticino Institute, Via Tesserete 48, CH-6900 Lugano, Switzerland

In recent years, the rate of pacemaker implantations has continued to rise throughout Europe [1]. In Switzerland, the number of pacemakers implanted has increased by more than 25% since 2015. A comparison between the implantation of pacemakers and cardiac resynchronization defibrillators (CRT- D) in Switzerland between 2015 and 2021 delineates some important trends in device therapy [2,3] (Figure 1). While the relative proportion of conventional single-chamber and dual-chamber pacemakers has remained relatively stable since 2015, a diversification of pacing therapies in terms of technologies and techniques has occurred. Specifically, a progressive increase in the number of leadless pacemakers has been observed, and an increased interest in the development of conduction system pacing (CSP) approaches, namely His-bundle pacing (HBP) and selective pacing of the left bundle branch (LBP), is emerging. Cardiac resynchronization therapy (CRT) devices have shown a slight decrease, particularly as far as implantations of CRT defibrillators are concerned. Overall, current trends in pacemaker implantation suggest the need for different technologies and device choices for the optimization of intra-procedural outcomes and the reduction in overall cardiac implantable electronic device (CIED)-related morbidity during follow-up, including pacemaker-related infections, lead malfunction, or chronic right ventricular pacing-induced cardiomyopathy. The scope of the present article is to supply further details on current trends in cardiac pacing, while addressing the main advances and challenges in this research field.

The increase in the rate of pacemaker implants is linked to different factors, including the aging of the population, increase in competent pacemaker implanting centres, and the remarkable rise and diffusion of transcatheter aortic valve implantation (TAVI) for the treatment severe aortic valve stenosis. About 10–20% of patients treated with TAVI require a permanent pacemaker after the procedure [4].

These patients are usually elderly, frail and present multiple co-morbidities. Post-procedural care implicates routine use of temporary pacing for 48 h after the procedure. In this immediate phase after TAVI, risk stratification for development of AV block remains a key issue for clinical decision-making [5]. Furthermore, for the prevention of pacing-induced heart failure during follow-up, particular consideration should be given to non-right ventricular apical pacing strategies in patients with pre-existing depressed left ventricular ejection fraction who develop AV conduction disturbance after TAVI [6].

Leadless pacemakers are among the possible options available for the treatment of elderly, clinically fragile patients who require a pacemaker. Different clinical needs the development of such leadless pacing technologies. First, delivering permanent pacing therapy through femoral venous access is particularly indicated in patients who present problems with axillary and subclavian vein accesses. Second, since leadless pacemakers constitute only 10% of the hardware and no pocket compared to the transvenous counterpart, pocket infection is completely eradicated, while intravascular infection risk is markedly reduced [7]. Third, long-term vascular-related complications, particularly chronic venous thrombotic occlusion, are also eliminated. Finally, a specific left ventricular endocardial leadless pacemaker system [8] overcomes limitations posed by unfavourable peripheral venous access or coronary sinus anatomy for the positioning of conventional coronary sinus leads in selected patients with heart failure and CRT indication.

Currently, the miniaturized leadless transcatheter pacing system is available both as a single-chamber pacing device (VVI(R) modality) [9] and a single-chamber device with atrial-sensing capability through accelerometer-based atrial detection (VDD modality) [10]. These systems significantly reduce peri-procedural complications, have a good long-term performance, and only rarely cause endovascular infection [7,9]. However, there are some concerns about procedural vascular access complications because of the bulky transvenous femoral sheath [11] and about approaches to device change and extractability [12].

As far as cardiac resynchronization therapy is concerned, changing trends in the implantation of CRT devices must be considered in the wider context of overall heart failure patient management improvements and specific developments in the field of device therapy. Early diagnosis, preventive strategies, and the recent adjunct of angiotensin receptor-neprilysin inhibitor and sodium-glucose cotransporter 2 inhibitors for the medical optimization of heart failure treatment [13], may potentially reduce the need for CRT. Although current trends in CRT device implantation remain relatively steady, reductions in CRT-D implantation have been observed (Figure 1). Non-device-associated improvements in heart failure management, the low rate of ventricular arrhythmic events in patients with non-ischemic heart disease [14], the lack of an accurate standardized approach for stratifying SCD in these patients [15,16], and long-term ICD-related morbidity, especially in the elderly patients, are all aspects that favour CRT-P over CRT-D. More recently, in selected patients, selective LBB pacing was found to be feasible and may correct and revert LBBB in selected patients [17], including heart failure patients with LBBB, and may represent a novel approach to deliver effective CRT [18]. However, the lack of data on the evolution of electrical parameters over time and on the extractability of these leads, that are fixed deeply into the interventricular septum, remain important concerns that hamper the diffusion of CSP [19]. There is a need for long-term multicentre data in the field of CSP.

An important area of development to address CIED-related morbidity is the clinical practice of transvenous lead extraction (TLE). The large prospective European registry ELECTRA has characterized the practice of TLE in Europe in terms of indications, techniques, peri-procedural outcomes as well as mid-term follow-up [20]. While the most common indications for TLE are either CIED-related infection or lead malfunction [20], the general tendency is now to avoid abandoning leads, especially in younger patients, for the prevention of future lead-related issues [21,22].

There is no doubt that cardiac pacing continues to play a central role in cardiology. The miniaturization of pacemakers and development of leadless pacing, the expansion of TLE practice, the reduction in the use of CRT-D, and the development of new techniques for CRT, all point in the direction of tailored pacing therapies with a particular concern for the reduction of intra-procedural and long-term CIED-related morbidity. The following factors will likely translate into overall reductions in CIED-related complications, shorter procedure times with less use of contrast dye and ionated radiation exposure, lower generator bulk in the pocket by implanting pacemaker devices instead of ICDs (or leadless pacemakers rather than the conventional transvenous kind), less intravascular leads, and less abandoned leads.

Through contributions from leading experts on cardiac pacing, CRT, and TLE, as part of the Special Issue entitled, “Advances in Cardiac Pacing and CRT”, the Journal of Clinical Medicine provides an updated view on the current state of cardiac pacing therapies, while providing valuable insights into future developments in this field.

## Figures and Tables

**Figure 1:**
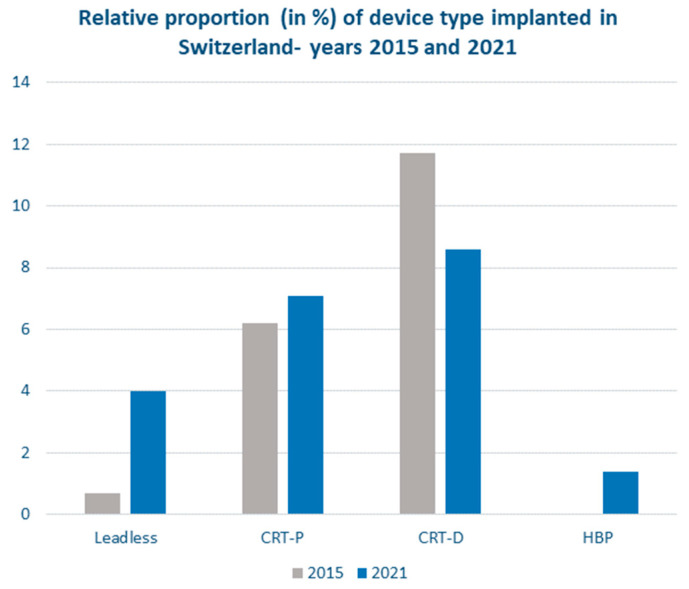
Relative proportion of CIEDs with pacing function implanted in Switzerland in 2015 (grey bars) and 2021 (blue bars). The proportions were calculated based on the total number of implanted devices with pacing function in 2015 (*n* = 6381) and 2021 (*n* = 8015), respectively.
